# Characterization of Nanodiamond-based anti-HIV drug Delivery to the Brain

**DOI:** 10.1038/s41598-017-16703-9

**Published:** 2018-01-25

**Authors:** Upal Roy, Vadym Drozd, Andriy Durygin, Jesse Rodriguez, Paul Barber, Venkata Atluri, Xiaohua Liu, Thomas G. Voss, Surendra Saxena, Madhavan Nair

**Affiliations:** 1 Department of Health and Biomedical Sciences, University of Texas Rio Grande Valley, Brownsville, Texas USA; 2 Center for the Study of Matter at Extreme Conditions, Florida International University, Miami, Florida USA; 3 Williams College, Williamstown, Massachusetts USA; 4 Department of Immunology, Institute of NeuroImmune Pharmacology, Center for Personalized Nanomedicine, Herbert Wertheim College of Medicine, Florida International University, Miami, Florida USA; 5 Proteomics Core, University of Texas Southwestern Medical Center, Dallas, Texas USA; 6 Vanderbilt Vaccine Center, Vanderbilt University Medical Center, Nashville, Tennessee USA

## Abstract

Human Immunodeficiency Virus Type 1 (HIV-1) remains one of the leading causes of death worldwide. Present combination antiretroviral therapy has substantially improved HIV-1 related pathology. However, delivery of therapeutic agents to the HIV reservoir organ like Central nervous system (CNS) remains a major challenge primarily due to the ineffective transmigration of drugs through Blood Brain Barrier (BBB). The recent advent of nanomedicine-based drug delivery has stimulated the development of innovative systems for drug delivery. In this regard, particular focus has been given to nanodiamond due to its natural biocompatibility and non-toxic nature–making it a more efficient drug carrier than other carbon-based materials. Considering its potential and importance, we have characterized unmodified and surface-modified (-COOH and -NH_2_) nanodiamond for its capacity to load the anti-HIV-1 drug efavirenz and cytotoxicity, *in vitro*. Overall, our study has established that unmodified nanodiamond conjugated drug formulation has significantly higher drug loading capacity than surface-modified nanodiamond with minimum toxicity. Further, this nanodrug formulation was characterized by its drug dissolution profile, transmigration through the BBB, and its therapeutic efficacy. The present biological characterizations provide a foundation for further study of *in-vivo* pharmacokinetics and pharmacodynamics of nanodiamond-based anti-HIV drugs.

## Introduction

HIV-1 infection remains one of the leading causes of mortality in the world. Although the development of combination antiretroviral therapy (cART) has significantly improved the mean lifespan of HIV-1 infected patients, the virus persists inside reservoir organs, such as the CNS and lymphoid tissues^1,2^. cART dosing regimens also show limitations based on distribution, metabolism, drug stability, and limited penetration into the CNS. This commonly results in suboptimal adherence, increased risk for treatment failure and development of viral resistance^3^. Moreover, drug characteristics are highly complex and differ substantially in chemical composition, molecular size, and protein binding. Therefore, drugs that provide a profound beneficial effect can also exhibit adverse side effect. Drug-induced toxicities and pharmacokinetic limitations commonly result in poor compliance and disease-related complications, including HIV-1 associated neurological disorders (HAND)^2,4^. HAND is perhaps the most common manifestation of HIV-1 pathogenesis that causes cognitive impairment and other CNS-related disorders^5–9^. Even with the advent of cART, over 40% of HIV-1 infected patients experience neurological complications^9^. Moreover, rates of HAND are likely to rise in the coming years as anti-HIV-1 therapies continue to extend the lifespan of patients. Currently, treatment options for HAND remain limited. Due to its poor penetration into the CNS, even potent antiretroviral therapy can only attenuate the progression of HAND. Eliminating HIV-1 reservoirs in the CNS will greatly increase the quality of life and lifespan of infected patients^10^. Therefore, the development of antiretroviral medicines to improve drug delivery into the CNS will continue to be an active and growing research field.

Nanomedicine-based drug delivery has revolutionized the targeted therapy field. With the help of nanotechnology, current therapeutic drugs can now be incorporated into a variety of biocompatible nanocarriers, thereby, improving their overall pharmacological properties. These formulations can then be further modified to deliver drugs to site-specific targets^11^ Presently, the most promising nanomaterial for drug delivery applications is nanocrystalline diamond or “Nanodiamond” (ND)^12^. The ND surface possesses an assortment of functional groups, most of which are oxygenated moieties, including carboxylic acid, lactone, ketone, ether, hydroxyl, etc. The natural biocompatibility, structural stability, and non-toxic nature of ND make it a widely applicable biomedical application^12–17^. Previous studies have demonstrated its capability as a drug carrier of doxorubicin, purvalanol A, 4-hydroxytamoxifen, and dexamethasone for human colon cancer, liver cancer, breast cancer and blood cancer therapies, respectively. Furthermore, these studies also indicated that ND possesses major cancer drug loading and sustained release capacities without producing any inflammatory reaction in human cells^18–22^. Previous mitochondrial function (MTT) and DNA fragmentation assays indicated that ND is not cytotoxic to many different human cell types^16^. Furthermore, ND has distinct advantages compared to other carbon-based nanomaterials, such as carbon nanotubes and nanographene, which have shown toxicity in many other studies. Moreover, they are not dispersed in water, which makes it difficult to use these excipients in nanoformulations^23–25^. On the contrary, the surface electrostatic potential of ND, which causes water to be drawn to surface, allows for the adsorption of drug molecules^13^. Therefore, ND provides an opportunity to use this material for anti-HIV-1 drugs that are not stable or dispersible in water. Considering its inherent characteristics, ND has been used for the delivery of many different therapeutic molecules, including drug, siRNA, hormones, proteins, and vitamins, without altering the biological activity of the molecule^13,26–31^. ND has the unique capability to solubilize water-insoluble drugs, making them more effective in repackaging hydrophobic anti-HIV-1 drugs. Finally, ND has also been shown to increase drug stability and prolong drug circulation time^32^.

In the present study, one cART drug, efavirenz (a non-nucleoside reverse transcriptase inhibitor or NNRTI, EFV), was formulated with unmodified ND or also referred as ND, -COOH surface modified ND (ND-COOH), and -NH_2_ surface modified ND (ND-NH_2_), respectively. As a free drug, EFV suffers from poor bioavailability due to blood plasma protein binding (99.5% binding)^33–35^. The small size and minimal toxicity of ND make the material an ideal candidate for improving drug delivery to the CNS. Furthermore, functionalized ND has the capacity to transport anti-HIV-1 drugs across the Blood-Brain Barrier (BBB). By applying ND towards drug delivery to treat HIV-1 CNS reservoirs, we have established that the conjugation of EFV with functionalized ND will substantially increase its therapeutic efficacy.

## Results

### Characterization of ND, ND-COOH, and ND-NH_2_

**Figure 1:**
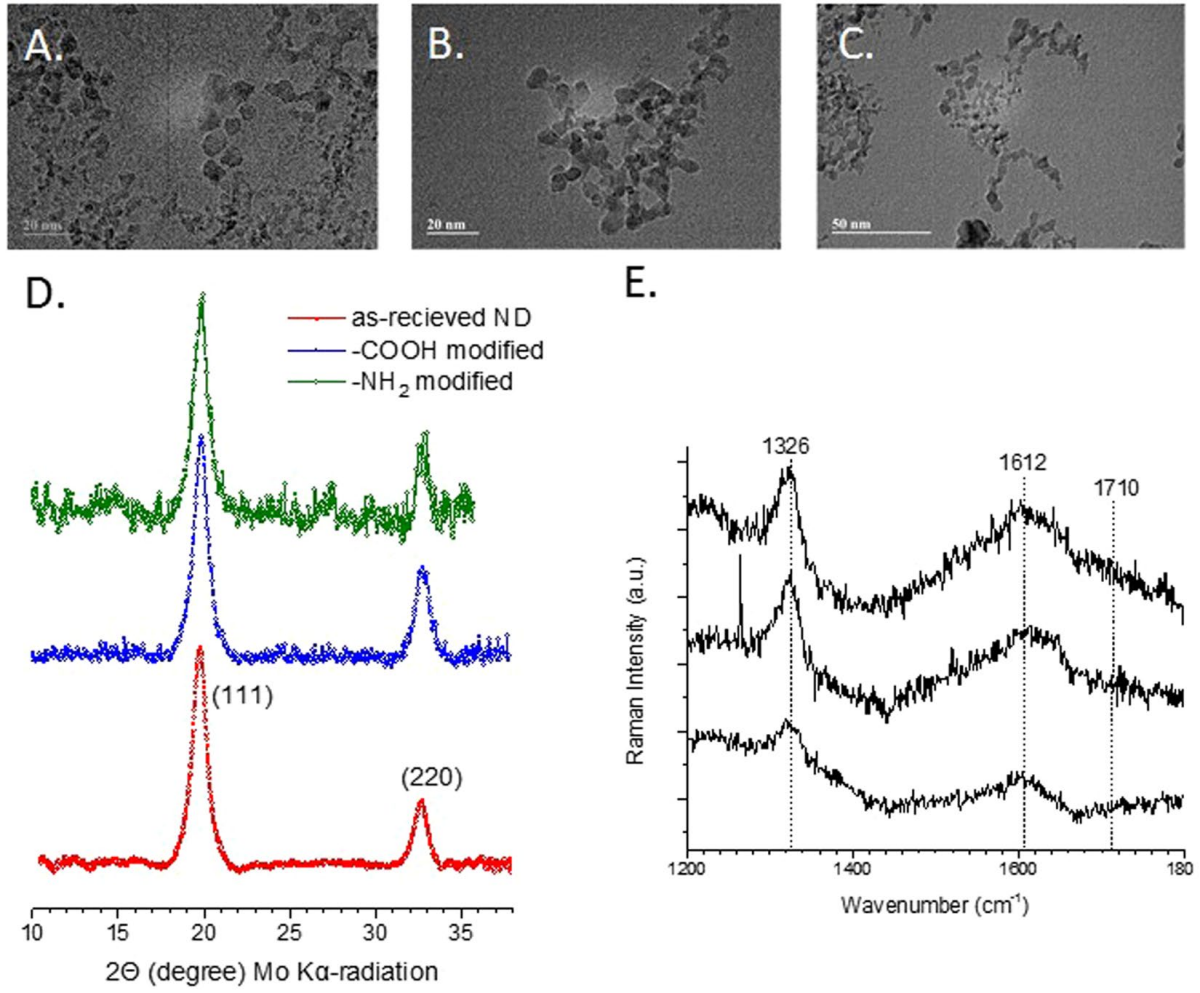
Characterization of ND: TEM images of (**A**) as-received ND, (**B**) –COOH and (**C**) –NH_2_ modified nanodiamonds. (**D**) Powder X-ray diffraction patterns of as-received ND in comparison with –COOH and –NH_2_ functional groups modified ND powders. The reflecting atomic planes of the diamond structure are denoted by Miller indices. (**E**) Raman spectra of (a) as-received ND, (b) –COOH and (c) –NH_2_ modified nanodiamonds.

To prepare the ND conjugated nanodrug, well-characterized ND was taken from different batches of ND powder prepared and characterized as per previously published protocol^36^. Figure 1 shows results of transmission electron microscopy (TEM), X-ray diffraction (XRD) and Raman spectroscopy characterization of nanodiamond powders. According to TEM, the average particle size of nanodiamond was 5 nm (3–6 nm as per manufacturer’s specifications) (Fig. 1A–C). The TEM images also showed that the primary diamond particles were partly agglomerated. Measured XRD pattern (Mo K_α_-radiation) of nanodiamond powder was a characteristic for good quality nanodiamonds. Two broad peaks correspond to diffraction from atomic planes with Miller indices (111) and (220) of the cubic diamond structure. Figure 1E shows Raman spectra of three ND that were used in this study: (a) unmodified, (b) –COOH surface  modified and (c) –NH_2_ surface modified surface modifications. Raman spectra were collected using Ar^+^ ion laser excitation (λ = 514.5 nm) and show vibrational feature at 1326 cm^−1^ which were a first-order diamond peak. The peak at 1619 cm^−1^ was a superposition of sp^2^ carbon (G-band) and OH groups on the surface. Both XRD and Raman study showed no graphitization of –COOH, -NH_2_ modified ND. Nonetheless, it was important to consider that the overall formulation should be nontoxic to neuronal cells to consider its potential for further study.

### ND-COOH induced more ROS compared to ND and ND-NH_2_ on SK-N-MC (ROS assay)

**Figure 2:**
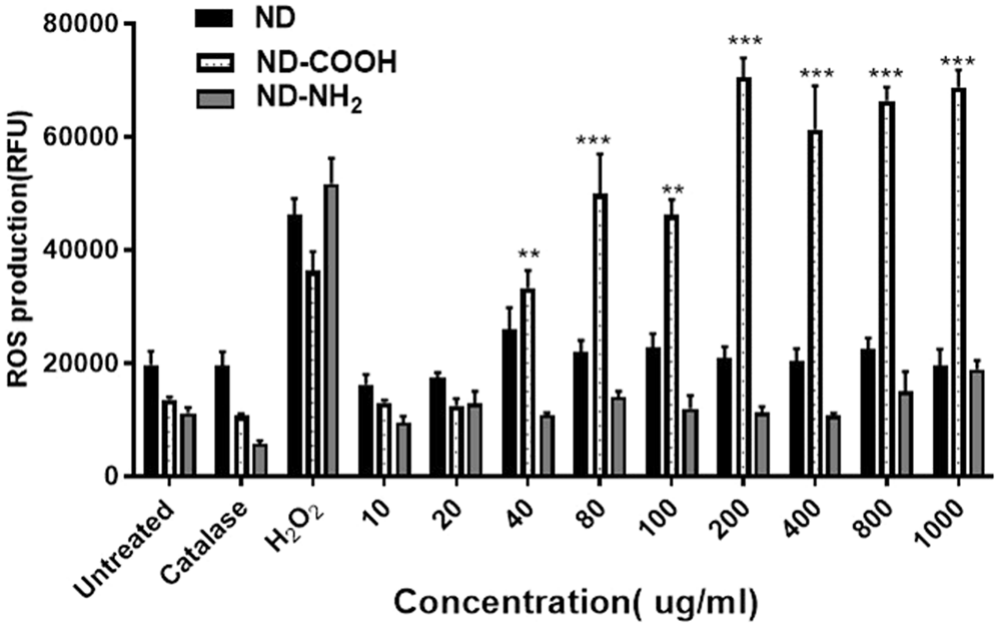
Effect of ND, ND-COOH, & ND-NH_2_ on ROS production on SK-N-MC cells: Three formulations were exposed at different concentrations (10–1000 *µ*g/ml) to SK-N-MC cells for 24 h. At the end of incubation, ROS production was measured in treated cells compared to untreated cells. The ROS production was measured in terms of mean ± SE relative fluorescence units (RFU) of eight independent experimental values. The statistical significance between untreated (ND) and ND-COOH treated cells were expressed as p values (**p < 0.001, ***p < 0.0003). There was no statistical significance between untreated (ND) and ND-NH_2_ groups.

In order to determine which formulation would be ideal for anti-HIV-1 drug delivery, it was important to observe whether any of these formulations by itself can induce ROS production to neuronal cells. In this regard, neuroblastoma cells (SK-N-MC) were treated with different concentrations of ND, ND-COOH, and ND-NH_2_, respectively, to observe the effect of these formulations on ROS production. As explained in the (Fig. 2), ND, ND-COOH, and ND-NH_2_ had a varied effect on SK-N-MC with respect to ROS production. Compared to antioxidant (catalase) and positive control (H_2_O_2_), ND and ND-NH_2_ formulation treatment did not exhibit any significant changes in ROS production at the different concentrations tested in this study. Whereas, ND-COOH treated cells showed significantly higher ROS release in all tested concentrations on SK-N-MC indicating overall cytotoxicity. Based on ROS assay, it was observed that ND-COOH formulation was substantially inducing more ROS production to SK-N-MC compared to ND and ND-NH_2_.

### ND was found to be nontoxic to SK-N-MC cells compared to ND-COOH and ND-NH_2_ (MTS assay)

**Figure 3:**
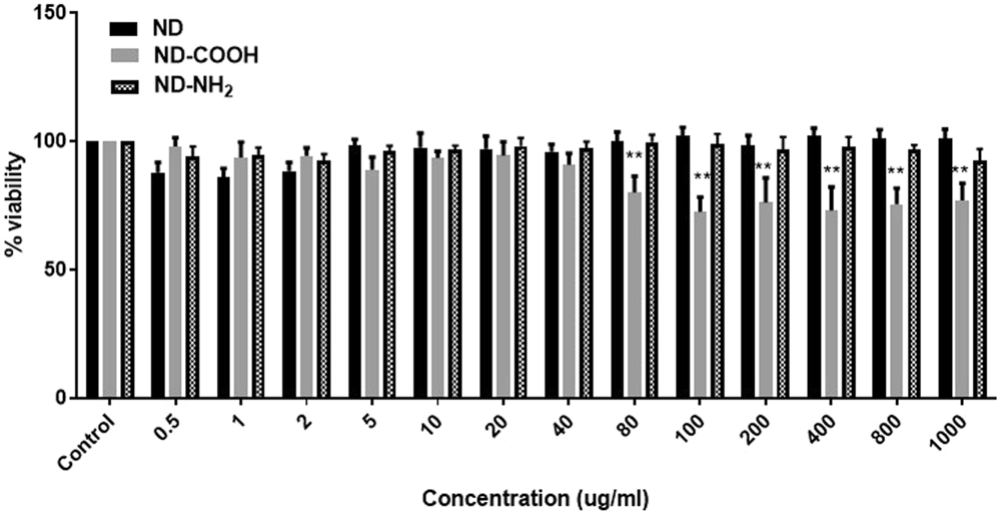
Cytotoxicity of ND, ND-COOH, and ND-NH_2_ on SK-N-MC cells. The cells were treated with a range of concentrations (0.5–1000 *µ*g/ml) for 24 h, separately. After incubation, MTS assay was performed and optical density (OD) was measured at 490nm. Graphical representation was made in terms of % survival of cells at different concentrations of three formulations corresponding to their OD values. Untreated cells (control) were considered as 100% viability and % survival was monitored based on control. The statistical significance between ND, ND-COOH, & ND-NH_2_ groups compared to control was expressed as p values (***p < 0.0001).

ND, ND-COOH, and ND-NH_2_ were also evaluated for their effect on the cell viability of SK-N-MC (Fig. 3). The formulations were introduced separately at different concentrations (0.5–1000 *µ*g/ml) to SK-N-MC and incubated for 24 h; MTS assay was then performed as per the manufacturer’s instruction (G3582, Promega, Madison, WI, USA). Figure 3 showed that SK-N-MC cell viability did not significantly change with increasing concentration of ND or ND-NH_2_ compared to control. Whereas, ND-COOH treatment had a significant effect on reducing cell viability at concentrations higher than 40 *µ*g/ml. Considering the chemical characterization, cytotoxicity, and ROS assay, it was observed that ND and ND-NH_2_ were more biocompatible and less toxic to neuronal cells.

### ND adsorbed comparatively more drug than ND-COOH

**Figure 4:**
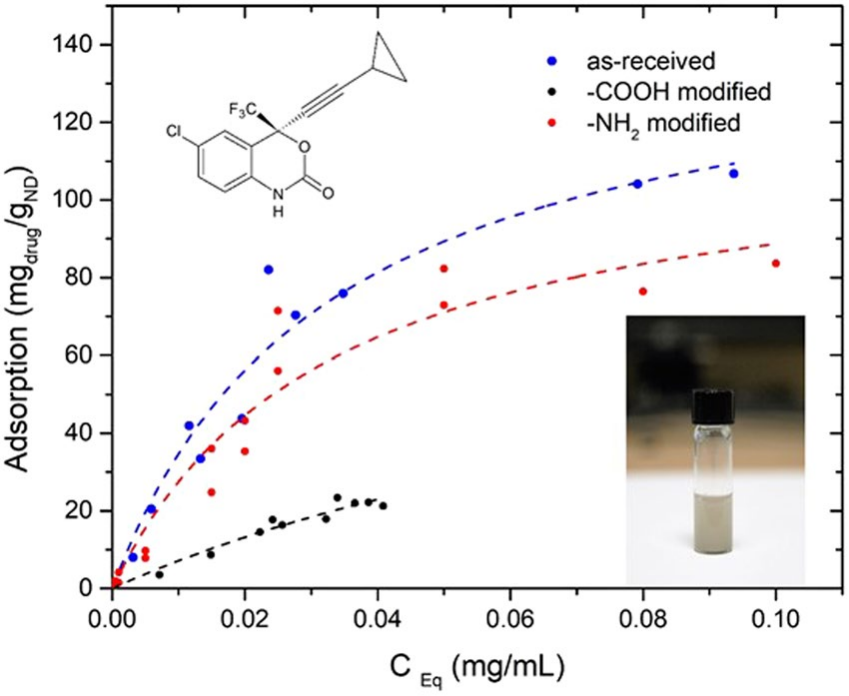
Adsorption isotherm of EFV on ND. The dotted line showed EFV adsorption by ND (blue), ND-NH_2_ (red) and ND-COOH (black), respectively. The adsorption values were calculated and represented as experimental points by Langmuir model. Inserts showed structural formula of efavirenz drug only and ND suspension in PBS buffer solution, respectively.

Adsorption isotherm of EFV on unmodified ND (milligrams of EFV absorbed per 1 g of ND) (in blue) as a function of C_eq_ (concentration of adsorbate in solution which is equilibrium with ND) is shown in (Fig. 4). The other formulations ND-NH_2_ (in red) and ND-COOH (in black) were also presented in Fig. 4 to demonstrate comparative adsorption of EFV by these formulations. Experimental points were fit by Langmuir adsorption model. This empirical model suggests homogeneous monolayer absorption of the drug on the surface of the adsorbent. Maximum adsorption capacity obtained from Langmuir isotherm is 161 mg EVF/g for unmodified ND. However, this value was not achieved experimentally due to the low solubility of EFV. KL constant which characterizes the bonding strength between the adsorbate (EFV) and the adsorbent (ND) is 23 mL/mg. In this regard, the relatively low value of KL suggested easy desorption of EFV from the ND. Comparative analysis of EFV adsorption on ND, ND-COOH, and ND-NH_2_, respectively, indicated that with respect to time and increasing concentration, there was quite a distinction between the three formulations. In Fig. 4, unmodified ND and ND-NH_2_ demonstrated a similar EFV adsorption compared to ND-COOH, which showed relatively low adsorption capacity for EFV at any given concentration and therefore not selected for further characterization in drug adsorption study. On the other hand, ND and ND-NH_2_ showed a similar capacity for drug loading and were nontoxic to cells. As ND-NH_2_ possessed a similar drug loading capacity and relatively similar biocompatibility as ND, therefore ND was selected for further *in vitro* characterization.

### Sustained release of EFV from ND-EFV (Dissolution study)

**Figure 5:**
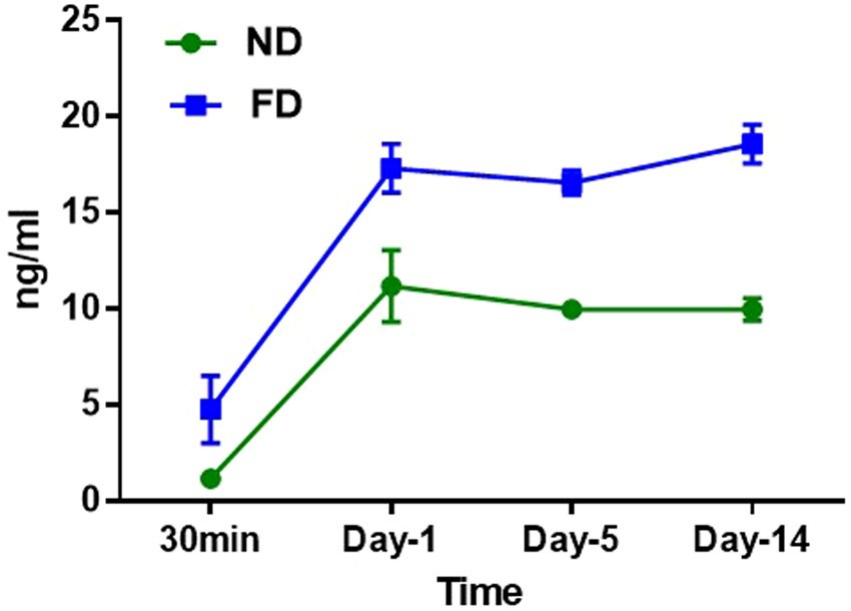
Drug dissolution study of nanodrug (ND) vs. Free drug (FD) *in vitro*. The released drug outside of dialysis bag was measured from 30 min up to day 14 by HPLC. The data indicated is an average of four different experiments (p > 0.05).

The sustained drug release profile of the ND-EFV formulation was determined in phosphate buffer saline (PBS) using equilibrium dialysis. The released drugs outside the dialysis bag were sampled at different time intervals (from 30 min up to 14 days) and measured by high-performance liquid chromatography (HPLC). As a positive control, equal amounts of the unformulated EFV (also called free drug or FD) were introduced directly to the *in vitro* PBS buffer, separately. Results were expressed as ng/ml of drug released from the ND relative to initial drug loading (Fig. 5). Compared to the FD (in blue), ND-EFV (in green) showed significant sustained drug release characteristics indicating improved pharmacokinetics of ND-EFV *in vitro*. This observation showed probable *in vivo* drug release pattern where a free drug is immediately released and get metabolized, whereas the ND-EFV sustained release pattern helps in CNS drug delivery where slow release of the drug is crucial for viral reservoir reduction. The overall chemical characterization of ND-EFV established a potential nanoformulation for anti-HIV drug carrier. Considering drug loading capacity of ND and possible cytotoxicity of ND-EFV at higher concentration, 40 ug/ml ND-EFV was used for further biological characterization.

### Drug release of ND-EFV *in vitro* environment through BBB

**Figure 6:**
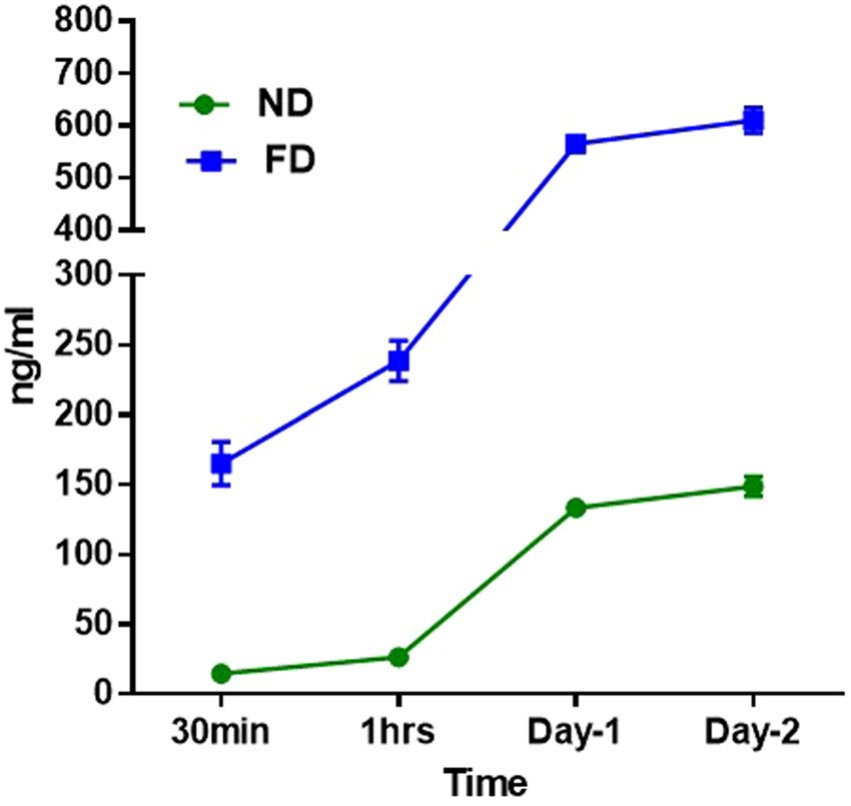
Drug delivery of ND-EFV through BBB *in vitro*. ND-EFV (green) and FD (blue) (40 *µ*g/ml) were introduced separately in the upper chamber of *in vitro* BBB model. EFV drug release was observed at different time points (30 min – Day 2) at the lower chamber of BBB model. A comparative analysis of sustained drug release from ND-EFV vs. FD through BBB was monitored with a drug content of the media with respect to time. Each set of drug release study was done in three replicates and data were represented with the statistical significance (p > 0.0001).

ND-EFV was further tested for its drug delivery capacity in crossing the BBB. An *in vitro* BBB model was set up to mimic the biological barrier that impacts drug delivery to the CNS. ND-EFV (40 ug/ml) was introduced into the upper chamber of the *in vitro* BBB model and drug content was monitored at the lower chamber as it crossed the other side of the BBB from 30 min up to day two. Unformulated EFV (40 g/ml) was also introduced in a separate setup, which served as a positive control. The BBB drug release study showed that unformulated EFV possesses a very robust drug release in  the brain side of the BBB model (Fig. 6). Nonetheless, ND-EFV specifically indicated significantly slower release of EFV compared to unformulated EFV. Thus, we have established that ND-EFV is a potential candidate for anti-HIV-1 drug delivery to the brain due to its ability to cross BBB and extend the retention time of EFV in the CNS.

### ND-EFV had no significant effect on neuronal plasticity

**Figure 7:**
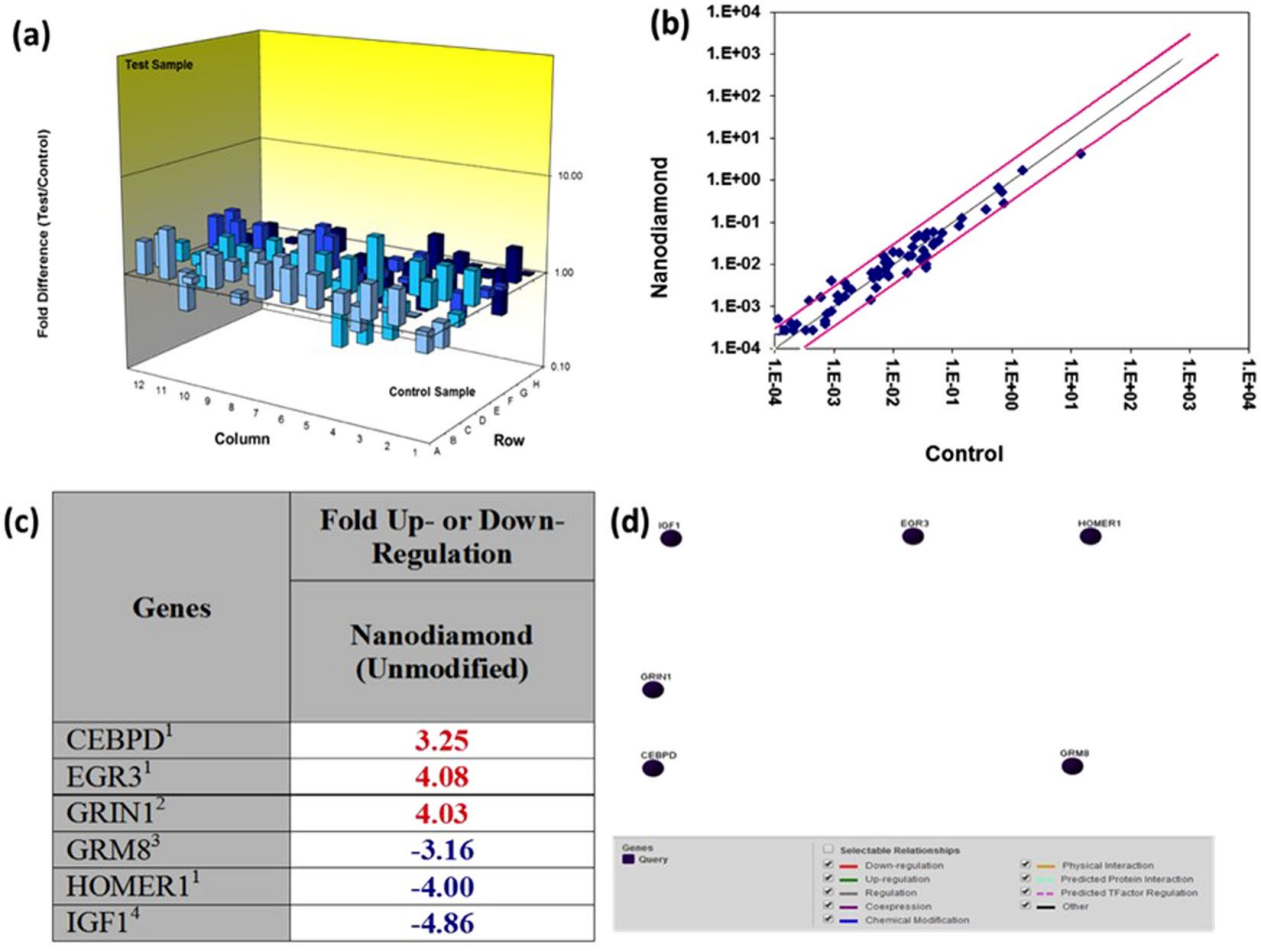
Human synaptic plasticity gene expression in ND-EFV exposed SK-N-MC cells. 84  genes analyzed that is related to the synaptic plasticity of the neurons through PCR array analysis. The only genes that significantly up (in red) /down (in blue) regulated (± ≥3 fold) are shown on the table. (**a**) 3D-profile of fold change of synaptic plasticity genes in ND exposed SK-N-MC cells. (**b**) Representative figures for scatter plot analysis of the changes in synaptic plasticity gene expression in ND-EFV exposed SK-N-MC cells: Spots associated with individual human synaptic plasticity gene were collected and converted into log_10_ scale. The central line indicates unchanged gene expression. The synaptic plasticity genes with expression levels higher or lower in treated neuronal cells than control cells are expected to produce dots that deviate from the centerline. The dots are allocated to positions that are above or below than the +3 fold or 3-fold line when the differences are greater than three folds. (**c**) Human synaptic plasticity genes expression in ND-EFV exposed SK-N-MC cells (fold change): Out of 84 genes analyzed, only genes significantly (± ≥ 3 fold) dysregulated were shown in this table (**d**) Gene-Gene interaction network for human synaptic plasticity genes dysregulated in ND-EFV exposed SK-N-MC cells.

In order to observe any deleterious effect of ND-EFV on neuron structural protein, we analyzed the expression of different synaptic plasticity genes in ND-EFV treated SK-N-MC as per published protocol^37,38^. Out of 84 human synaptic plasticity genes, we have observed significant up-regulation of three synaptic plasticity genes (CEBPD, EGR3, and GRIN1) in ND treated neuronal cells compared to control cells. We also observed significant down-regulation of GRM8 (3 fold), HOMER1 (4 fold), and IGF1 (4.8 fold) gene when compared to the untreated control cells (Fig. 7). Nonetheless, Fig. 7(d) gene-gene interaction analysis illustrated that all the genes that are affected by ND-EFV exposure are not directly related to each other with respect to their functional activity. Therefore, there was no connection between up-regulated or down-regulated genes. This observation over all indicated that there is no potential deleterious effect on neuronal plasticity of SK-N-MC in presence of ND-EFV. This finding also opens up a potential for the long-term use of ND-EFV without any side effects on human neurons.

### Therapeutic efficacy study of ND-EFV

**Figure 8:**
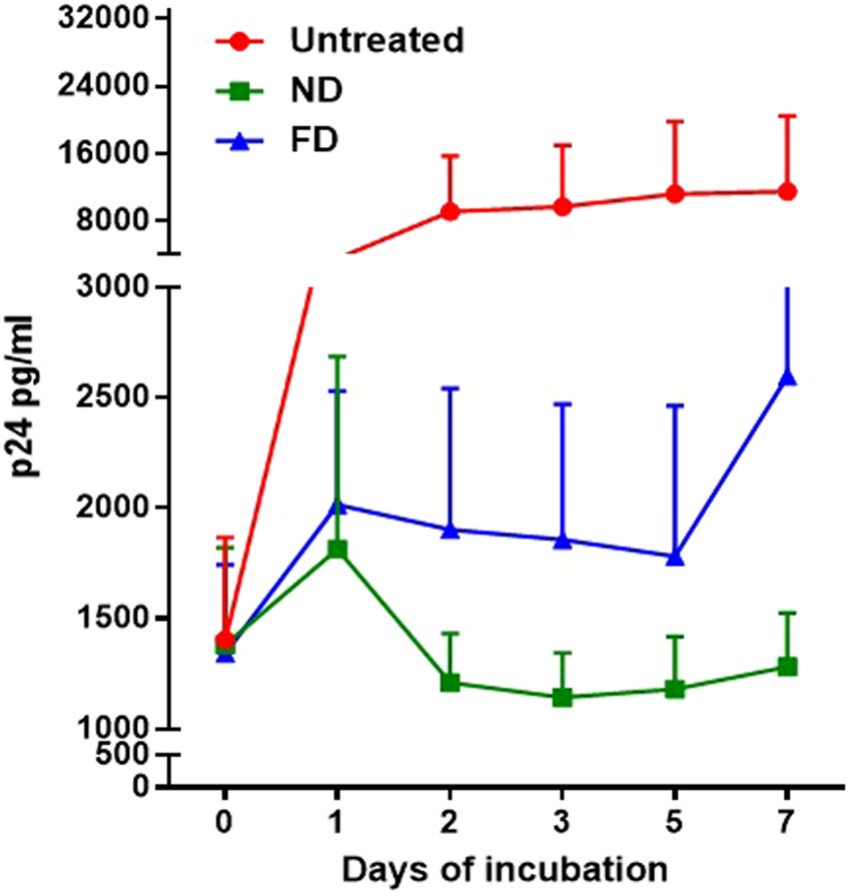
Therapeutic efficacy of ND-EFV on HIV-1 infected Macrophages. HIV replication was monitored in three different conditions. The HIV-infected cells that were kept untreated served as positive control (in red). HIV-infected cells were treated with unformulated EFV (40 *µ*g/ml) served as reference (in blue). The ND-EFV (40 *µ*g/ml) treated cells were considered as a test (in green). In all three sets of treatments, the p24 level was monitored at a different time interval to measure the effect of treatment on HIV replication. In this case, 24h post infection was considered as day 0. Statistical significance was calculated with respect to *p* values (*p* < 0.0001).

Anti-HIV-1 efficacy of ND-EFV was tested by introducing the formulation in HIV-1 infected primary human macrophages *in vitro* condition. Initially, Human peripheral blood mononuclear cells (PBMC) were isolated and incubated to be differentiated to macrophages. Following the differentiation, cells were infected with HIV and FD and ND-EFV were introduced separately to HIV-1 infected macrophages. HIV-1 p24 level at cell supernatant of drug-treated cells indicated a significant difference on viral inhibition by unformulated EFV and ND-EFV. The effect of ND-EFV on HIV-1 replication was observed to be very effective and sustained over a period of seven days. In contrast, the unformulated EFV could control viral replication up to day five, thereafter, p24 returned to untreated levels. Infected and untreated macrophages were kept as positive controls. Overall representation in Fig. 8 confirmed the efficacy of ND-EFV over HIV-1 replication compared to unformulated EFV. This further demonstrated the promise of using ND-EFV for targeted drug delivery towards the CNS. Nonetheless, the present study did not confirm the direct CNS drug delivery of ND-EFV and possible consequences. In future, this formulation will be used to conjugate with a specific targeting agent (antibody, ligand, etc.) for direct drug delivery of anti-HIV-1 drugs to the CNS.

## Discussion

EFV is one of the very effective NNRTIs that has been in use in present combined antiretroviral therapy (cART) for the treatment of HIV-1 infection. However, its low bioavailability, poor pharmacokinetics, and subsequent drug resistance have made it necessary to optimize therapeutic delivery agents to make EFV more targeted and effective^39–41^. Among the many nanocarbon materials that have been investigated for use as drug carriers, ND, is the most promising due to its many favorable properties, including, chemical inertness, biocompatibility, and easy availability compared to other nanocarbon materials^17^.

According to TEM study (Fig. 1A–C), nanodiamond particles have a spherical morphology and average particle size in a range of 5–6 nm that is close to vendor’s specifications (3–5 nm). As it should be expected, modifications of ND surface did not alter ND particles size, morphology or crystal structure (Fig. 1D). Raman spectra of all samples display the characteristic feature of the diamond phase (peak at 1326 cm^−1^). According to Mochalin *et al*., (2008) a broad feature around 1612 cm^−1^ can be assigned to graphitic carbon on ND surface (the G-band) with some contribution from hydroxyl groups that are chemo- or physisorbed onto the surface^42^. Considering the fact that cytotoxicity was one of the deciding factors for screening the right drug formulation for the neuronal cells, ROS and MTS assay was performed with all three formulations. As the neuronal cells are very sensitive to ROS, it was important to screen a formulation that does not induce significant amount of ROS production in the neuronal cells^43^. ROS study explained that ND-COOH can induce more ROS production than ND or ND-NH_2_.Further study on cytotoxicity effect of all three formulations also established that ND-COOH was significantly toxic at a higher concentration above 40ug/ml. Following the surface modification and cell cytotoxicity study, ND, ND-COOH and ND-NH_2_ were loaded with EFV as per the protocol mentioned in the method. The EFV adsorption study on ND, ND-COOH, and ND-NH_2_ clearly indicated the potential of ND and ND-NH_2_ formulation over ND-COOH based on Lingmuir adsorption model. This observation also established the potential of ND and ND-NH_2_ over ND-COOH. Moreover, the dynamic stability of surface modified and unmodified ND were characterized in sterile phosphate buffer saline (PBS), DMSO and in combination. Presently, the suspension stability was evaluated by visual observation in this study. Surface modified ND in these experiments had lower stability compared to unmodified ND. Unmodified ND was found to be stable for several hours in PBS suspensions and for several days in DMSO suspension (unpublished data). Experiments on further stability of ND/EFV suspensions is in progress and will be reported elsewhere. In the present study, ND was suspended in 1:9 DMSO-PBS (volume/volume) for chemical characterization. The objective of the study was to find a formulation that is nontoxic to neuronal cells and effective against HIV. In this regard, ND was found to be an ideal formulation because ND-COOH showed cytotoxicity and ND-NH_2_ had similar drug loading as ND. Considering all these factors, ND was selected for further characterization *in vitro*. In the dissolution study, ND-EFV demonstrated an extended release of EFV from drug formulation up to fourteen days indicating much more stable formulation than FD. The possible mechanism of FD release delay could be caused by sustained drug release from the reservoir effect in polymeric vehicles, which may attenuate with time and another retention effect from the dialysis bag itself. It is already reported that the dialysis bag may affect the *in vitro* drug release^44^. Therefore, even considering this delay impact from dialysis bag, the dissolution profile of ND-EFV was still observed to be adequate for sustained drug release requirement. EFV drug release study in BBB model also explained the potential benefit of ND-EFV over FD with respect to higher drug retention and delivery capacity to the brain which is one of the major impediments of targeted drug delivery. In this regard, it is worth mentioning that *in vitro* BBB membrane also present a concentration difference of FD between upper and lower chamber of the BBB model that might influence the faster release of FD to the media at the lower chamber. In addition, previous studies have also established the unformulated EFV exposure can disrupt the integrity of BBB and possible toxicity to BBB endothelial cells^40,41^. This might also allow more drug to pass through the BBB in a shorter period of time. The robust release of FD in the brain can also cause neurotoxicity and will get metabolized much faster upon release. Whereas, the sustained release of drug from nanoformulation will provide a constant control on viral replication in the brain with limited toxicity^11^. Since ND-EFV has demonstrated the stability of the biological environment and extended release of EFV crossing BBB, it provided a strong evidence to use this nanoformulation for anti-HIV drug development.

The present study also investigated the direct effect of ND-EFV on the expression of eighty-four key genes of neurons that are related to synaptic alteration during learning and memories. Alteration of these genes has a direct effect on some of the neuronal events like long-term potentiation (LTP), Long-term depression (LTD) etc. and those could be few potential side effects of ND-EFV. The synaptic plasticity gene array showed that there was no immediate side effect of ND-EFV on neuronal cells. This was also an important observation since unformulated EFV has toxic effects on neurons and brain cells^40,41^. The therapeutic efficacy study also indicated that FD had an immediate effect on HIV replication. However, its efficacy significantly decreased within five days. On the other hand, ND-EFV could control viral replication up to seven days and beyond establishing further the suitability of ND-EFV for long-term anti-HIV therapy.

The investigation of ND towards drug delivery and imaging has only begun very recently. Indeed, to our knowledge, the present study has shown for the first time the application of ND in targeted anti-HIV-1 drug delivery towards the CNS. Our study has established that ND-based nanodrugs are nontoxic to the CNS and specific molecular characterizations of these nanodrugs can cross the BBB to deliver drugs to the CNS. Most importantly, the initial chemical characterization of ND and the following biological characterization of ND-EFV has revealed a promising candidate for nanodrug delivery of anti-HIV-1 drugs to the CNS. However, the present study could not confirm the fate of the ND after EFV release since it was done in *in vitro* microenvironment. In the future, further *in vivo* study on ND-EFV will be performed to maximize its potential use as a therapeutic agent for HIV-1 patients and its detailed metabolomics.

## Methods

### Cell culture and reagents

Human neuroblastoma cells (SK-N-MC) (ATCC Cat # HTB-10) were cultured in Eagle’s minimum essential medium (MEM) (ATCC catalog # 30–2003) supplemented with fetal bovine serum to a final concentration of 10% (ATCC catalog # 30–2020). These cells and media were obtained from American Type Culture Collection, Manassas, VA and 1% antibiotic and antimycotic solution (catalog # A5955) was obtained from Sigma-Aldrich, St. Louis, MO. HIV-1 Ba-L (clade B) (Cat. # 510) was obtained through AIDS Research and Reference Reagent Program, Division of AIDS, NIAID, NIH. ND powder (3–6 nm, purity 97 + %) was purchased from Nanostructured and Amorphous Materials Inc. (Garland, TX, USA). EFV drug powder and all other chemicals were obtained from Sigma-Aldrich (St. Louis, MO, USA). An Agilent 1200 HPLC system (Palo Alto, CA) coupled to an Applied Biosystem 4000 Q TRAP quadrupole linear ion trap hybrid mass spectrometer (Applied Biosystems/MDS Sciex, Foster City, CA) was used for drug analysis. The HPLC-MSMS system is controlled by ChemStation and Analyst 1.4.2 software, respectively. All chromatographic separations were performed on an Agilent ZORBAX RP 18 column (3.5 *µ*, 150 mm × 0.5 mm) (Palo Alto, CA).

### Characterization of nanodrug

Unmodified nanodiamond powder (ND) was used as it was for this study. Amine-functionalized nanodiamond (ND-NH_2_) was fabricated by the procedure reported^45^. 50 mg ND powder was dispersed in absolute ethanol via sonication for 30 min, and then, an excess solution of APTES was slowly dropped and stirred overnight at refluxing conditions on a water bath. The ND-NH_2_ was centrifuged and subsequently washed with ethanol for at least 5 times, which was then dried at 80 °C under vacuum for 12 h. Oxidation (surface modification with carboxyl-groups) was performed at 425 °C in air for 2 h^46^.

### Transition electron microscopy (TEM)

The preliminary characterization of the nanodrugs was performed using a Phillips CM-200 200 kV Transmission Electron Microscope (TEM) with Energy Dispersive Spectroscopy (EDS) housed at FIU’s Advanced Materials Engineering Research Institute (AMERI). TEM observation was helpful for determining the particle size, morphology and dispersity of the nanodrugs.

### X-ray diffraction (XRD)

An X -ray diffraction study of ND was performed using a Bruker GADD/D8 X-Ray diffraction system with Apex Smart CCD and imaging plate detectors and direct-drive rotating anode. The MacSci rotating anode (Molybdenum) operates at 50 kV voltages and 20 mA current. The 2D diffraction patterns obtained were integrated using Fit2D software^47^. This X-ray diffraction method was used for structural characterization of the materials, particle size measurements of the crystalline phases, and for estimating the degree of crystallinity.

### Raman spectroscopy characterization

A continuous wave (CW) argon ion (Ar+) laser (model 177G02, Spectra Physics) of 514.5 nm in wavelength was used as a source of monochromatic radiation. Backscattered Raman spectra were collected by a high-throughput holographic imaging spectrograph (Model HoloSpec f/1.8i, Kaiser Optical Systems) with volume transmission gratings, a holographic notch filter, and a thermoelectrically cooled charge-coupled device CCD detector (Andor Technology). The Raman system has a spectral resolution of 4 cm^−1^. The spectra were usually collected with 10 min exposure.

### Reactive Oxygen Species (ROS) assay

ROS productions in SK-N-MC following exposure to different concentrations of ND, ND-COOH, and ND-NH_2_ were detected using dichlorofluorescein diacetate assay (DCF-DA; Molecular Probes, Eugene, OR) as per previously published protocol^48^. Cells were cultured in 96-well plates (100,000 cells well) overnight to allow 70% confluence. The next day, cells were treated with different concentrations of ND, ND-COOH, ND-NH_2_ (10, 20, 40, 80, 100, 200, 400, 800, and 1000 *µ*g/ml) for 24 h, respectively. The following day, cells were washed and pretreated with antioxidant, catalase (0.001 mg) for 2 h. After incubation, the cells were treated with DCF-DA (100uM) for 1 h at 37 °C, and finally, the cells were read in a BioTek Synergy HT microplate reader (excitation 485 nm and emission 528 nm; BioTek, Winooski, VT). Cells treated with H_2_O_2_ (50 *µ*M) for 2 h were included as a positive control.

### Cellular toxicity of nanodrug

Cytotoxicity of ND, ND-COOH, and ND-NH_2_ on SK-N-MC cells was determined via MTS assay (G3582, Promega, Madison, WI, USA)^44^. Cells were pre-incubated in 96-well plates with SK-N-MC cells and then treated with various concentrations of nanodrug (control, 0.5, 1, 2, 5, 10, 20, 40, 80, 100, 200, 400, 800 and 1000 *µ*g/ml) for 24 h at 37 °C. After treatment, cells were washed and incubated with fresh respective growth medium. Cells were further incubated with 20 *µ*l of MTS reagent (CellTiter^96®^ Aqueous One Solution, Madison, WI, USA) in complete 100 *µ*l cell media for 1 h at 37 °C. Following incubation, an absorbance at 490 nm was measured using the BioTek plate reader (BioTek, Winooski, VT, USA). Untreated cells incubated with fresh media were used as a negative control. All measurements were taken as a mean of eight independent experimental values. The net absorbance (A) was taken as an index of cell viability. The cell viability was calculated as sample/control ×100%. The nanoformulations that did not cause more than 10% loss in cell viability after at least 24 hr exposure were considered nontoxic.

### Drug adsorption study

In order to make the initial calibration curve, concentrations of 1:9 DMSO-PBS and EFV were made at 5, 40, 50, and 100 *µ*g/ml from 10 mg/ml stock solution of EFV in a total volume of 2 ml for each concentration. The initial calibration curve was created using the absorbance values of the corresponding concentrations (250 nm). The dilutions were thoroughly mixed and the optical density (OD) value of each concentration was measured twice using Hitachi U-2910 Spectrophotometer (UV Spec.) 2 mg of ND was then added to each glass tube. The tubes were then sonicated in an ultrasonic bath for 2 min. The tubes were then shaken at 25 °C and 190 rpm for approximately 24 h. The contents were then transferred to microfuge tubes and mixed using Eppendorf Thermomixer for 1h at 600 rpm. The solutions were then centrifuged at 13,000 rpm for 1 h. The supernatant of the solutions was removed (conservatively), leaving the ND pellet. The OD values of the supernatant for each concentration were measured using Hitachi U-2910. Based on the measured absorbance values before and after the adsorption, the concentrations of drug in the resulting solutions were calculated using the measured OD values.

### Drug analysis through HPLC-MS/MS

The EFV concentration in biological samples was quantitated by a previously reported Selected reaction monitoring (SRM) method^49^ In brief; the samples were homogenized with deionized H_2_O at a ratio of 1:2 (w/v). 1 mL of ice-cold acetonitrile was added to a 100 *µ*L homogenized samples which spiked with 10 *µ*L IS (2.0 g/mL lopinavir). The sample was then vortexed for 3 min, shaken for 15 min, and centrifuged at 16,000 × g for 10 min. The supernatant was aspirated, dried under vacuum at room temperature, and dissolved in 100 *µ*L 50% methanol aqueous solvent. Before injection, the sample was centrifuged for 10 min at 16,000 g, 8 *µ*L of supernatant was injected into the HPLC–MS/MS system for analysis. For all samples, the final concentration of IS was 200 ng/mL. The linearity of the method (in the range of 0.2–1000 ng/mL) was confirmed by preparing 3 standard curves on 3 different days.

### Dissolution study of the ND-EFV

ND-EFV was tested for long-term sustained release capacity in physiological buffer media. Briefly, a solution of ND-EFV nanoparticles (0.5 ml, concentration: 2 mg/20 mg ratio drug/ND) was placed into a dialysis bag (molecular cutoff: 6 kDa), sealed, and put into a tube filled with 30–40 ml dissolution solution (composition: 0.1% Tween 20 PBS with pH 7.4). This concentration was decided based on the initial standardization of drug loading on ND through adsorption study (unpublished data). Dispersion of the ND-EFV in PBS (pH 7.4) was placed in the dialysis bag (D-bag) (Pur-A-Lyzer Maxi Dialysis Kit, SIGMA) and dialyzed against the respective buffer solution at 37 °C at a speed of 150 rpm. At each time (30 min, day-1, day-5 and day-14), 100 *µ*l of the solution was taken out from the tank and the equal amount of fresh buffer was replenished. The free EFV release study was also measured in similar conditions simultaneously. Final drug concentrations of the collected samples were determined by HPLC as mentioned above.

### Human synaptic plasticity RT^2^ profile PCR array

Synaptic plasticity gene expression profiling was done in SK-N-MC control (untreated) cells and ND-EFV (40ug/ml) treated cells for 24h using 96 well format RT2 Profile PCR Array Human Synaptic Plasticity kit (SABiosciences, Cat. # PAHS-126Z) using Stratagene Mx3000p qRTPCR instrument^37,38^. This test includes 84 diverse genes important in human synaptic plasticity, including Immediate-Early Response (n = 30), Late Response (n = 2), Long-Term Potentiation (n = 28), Long-Term Depression (n = 21), Cell Adhesion (n = 9), Extracellular Matrix & Proteolytic Processing (n = 5), CREB Cofactors (n = 10), Neuronal Receptors (n = 19), Postsynaptic Density (n = 15), as well as other genes involved in the synaptic plasticity (n = 2). Relative abundance of each mRNA species was assessed using RT^2^ SYBR Green/ROX PCR Master mix (SABiosciences, Cat # 330520) and aliquoted in equal volumes (25 *µ*l) to each well of the real-time PCR arrays. The real-time PCR cycling program (as indicated by the manufacturer) was run on a Stratagene Mx3000p qRT-PCR thermal cycler. The threshold cycle (Ct) of each gene was determined by using the Stratagene MaxPro software. CT data was uploaded into the data analysis template on the manufacturer’s website (http://pcrdataanalysis.sabiosciences.com/pcr/arrayanalysis.php). The relative expression of each gene in each ND-EFV treated group was compared with the expression in control cells and it was calculated on the website using the ∆∆ CT method with five housekeeping genes as controls. Controls are also included on each array for genomic DNA contamination, RNA quality, and general PCR performance. The result was represented in fold change in the expression in each gene of test and compared with control gene incorporating the statistical significance among the tested gene expression in the table form. The data also represented in 3D plot profile, scatter plot and volcano plot along with tabular form of up or down regulated gene expression. A gene-gene interactive analysis was also presented based on the level of expression including up & down regulation, co-expression, chemical modification and other protein related interaction. A direct gene-gene interaction would have indicated by direct joined lines between two or more genes in the plot and no gene-gene interaction was indicated as no interconnecting lines between different genes spots within the plot. This observation presents the valuable information about functional co-relation of different up and down regulated genes with respect to their function related to neuroplasticity.

### Drug delivery through BBB

Primary Human Brain Microvascular Endothelial Cells (HBMVEC) and astrocytes were obtained from Science Research Laboratories, Carlsbad, CA. *In vitro* BBB model was established in the trans-well plate as per published protocol^50,51^. HBMVEC was inoculated at the upper side of 0.4 um pore size PTFE membrane tissue culture inserts (Corning, NY) at an initial concentration of 10^4^ cells/well. A confluent layer of human astrocytes was grown on the lower side of the membrane. After incubation, the integrity of the BBB was measured with transendothelial electrical resistance (TEER) using Millicell ERS microelectrodes (Millipore, Billerica, MA, USA). The typical TEER values of untreated BBB were observed to be around ~200 Ω/cm^2^. For the drug delivery study, HBMVEC were allowed to grow up to 70% confluency and then ND-EFV (40 *µ*g/ml) was introduced into the upper chamber of the transwell insert. Following that, media was collected at different time points (30 min, 1 h, Day-1, and Day-2) from the lower chamber and replenished with fresh media. The drug content of the collected media was measured by HPLC as mentioned above. Simultaneously, the unformulated drug was also introduced in a similar setup and monitored for drug release with respect to the time period.

### Therapeutic efficacy

ND-EFV was investigated for anti-HIV-1 efficacy in the primary human macrophage as previously published protocol^48^. Human PBMC were obtained from a healthy subject through purchase from a local clinic and differentiated into macrophages as previously published^49^. PBMC were isolated with Ficoll-Hypaque (Pharmacia) gradient and cells were incubated for differentiation for 7 days in the presence of human macrophage colony stimulating factor (MCSF, Sigma) to macrophages. Following incubation, macrophages were infected with HIV-1 1Ba-L (NIH AIDS research and reference reagent program Cat# 510) (100 ng/ml) for 24 h. Following 24 h of infection (day 0), cells were washed to get rid of any unattached virus and fresh media was added. At the same time, 40 *µ*g/ml of ND-EFV and FD was added separately to these cells in two different setups. The HIV-1 infected macrophages served as a positive control. The ND-EFV/ FD-treated cells were monitored for up to 10 days along with infected control cells. The cell supernatant was collected at day 0, 1, 2, 3, 5, and 7 days post-treatment. The viral p24 level in the supernatant was measured with p24 ELISA assay (Cat. # 0801200, Zeptometrix, USA). The p24 level at different time points provided the level of viral inhibition caused by ND-EFV and FD. The p24 level at the culture supernatant will be inversely proportional to the therapeutic efficacy of ND-EFV.

### Statistical analysis

Experiments were performed in multiple replicates and the data were presented as mean ± SEM. The statistical significance of each experiment was analyzed by two-tailed paired t-test and with one & two-way ANOVA test with GraphPad Prism software (GraphPad Prism Software Inc. San Diego, CA) and a p-value of ≤0.05 was considered as significant.
